# Diagnosis and Management of Pediatric Blunt Cerebrovascular Injuries: A Narrative Review

**DOI:** 10.3390/jcm15114069

**Published:** 2026-05-25

**Authors:** Ania Murillo, Nelson V. Guevara, Nicholas J. Iglesias, Daniel M. Alligood, Eduardo A. Perez, Carlos T. Huerta

**Affiliations:** 1 Miller School of Medicine, University of Miami, Miami, FL 33136, USA; 2 Division of Pediatric Surgery, DeWitt Daughtry Family Department of Surgery, University of Miami, Miami, FL 33136, USA

**Keywords:** pediatric, trauma, blunt cerebrovascular injury, BCVI, stroke

## Abstract

**Background/Objectives:** While standardized guidelines have been established for the evaluation and management of blunt cerebrovascular injuries (BCVIs) in adults, there remains a paucity of standardized guidelines for BCVIs in pediatric populations. Shortcomings in treatment algorithms also persist as uncertainty remains about the optimal approach to manage these cases. A review of the literature was performed to compile the current evidence and provide recommendations based on current overarching trends. **Methods:** PubMed was queried for studies related to the diagnosis and management of BCVIs in the pediatric population. Prevalence, mechanism of injury (MOI), screening criteria, diagnostic modality, vascular injuries identified, associated injuries, treatment, and patient risk factors were analyzed. **Results**: The Utah and McGovern criteria were the first tools developed for screening BCVIs in pediatric patients. Among all screening tools, the high sensitivity and specificity of the McGovern criteria support its use as the optimal screening strategy to date for pediatric patients. Given the high prevalence of high-energy MOI, observation is the most common approach chosen due to contraindications to medical therapy. Antiplatelet agents showed no significant differences in stroke prevention or hemorrhagic complications compared to anticoagulation. Strokes represent the primary source of morbidity among pediatric patients with BCVIs. **Conclusions**: Pediatric BCVIs represent an uncommon but clinically significant consequence of blunt trauma, with a significant risk for ischemic stroke and neurologic morbidity. Early recognition through appropriate screening with pediatric-specific screening criteria, CTA imaging, and timely initiation of grade-based treatment can help mitigate injury progression and complications.

## 1. Introduction

While standardized guidelines have been established for the evaluation and management of BCVIs among adults, their dissemination in pediatric populations remains limited. Given the heterogeneous approach to blunt cervical trauma in the pediatric population, there remains a significant proportion of BCVIs that go undetected and therefore untreated in children [1]. In one study assessing the management of pediatric BCVIs in six level-one trauma centers, only 42.5% of patients received appropriate imaging after meeting the Memphis screening criteria [2]. Therefore, the prevalence of BCVIs in the pediatric population likely remains ill-defined and warrants further evaluation to better understand the burden of these injuries in children.

The current guidelines for BCVI screening in the adult population include the Eastern Association for the Surgery of Trauma (EAST) guidelines, last updated in 2010, and the Denver criteria, last updated in 2012 [3,4]. While these criteria have not been well studied in pediatric populations, available evidence suggests that applying adult screening criteria to children lacks specificity, leading to increased imaging and unnecessary radiation exposure [5]. To improve the screening and diagnosis of BCVIs in the pediatric population, various institutions have implemented new screening criteria tailored to this population. The Utah score and McGovern score have recently been published, in 2017 and 2018 respectively, to enhance diagnostic accuracy and improve overall patient outcomes [6,7]. Despite the emergence of recently published screening criteria, a consensus has yet to be established, as evidenced by the continued absence of a clearly defined gold standard for screening. Further investigation of the prevalence, risk factors, management, and outcomes associated with BCVIs is imperative to optimize the care of pediatric trauma patients.

Shortcomings in treatment algorithms persist, as uncertainty remains about the proper approach to managing these cases. These inconsistencies in the current literature have left a majority of children diagnosed with BCVIs untreated or inadequately treated, further highlighting the need to develop appropriate management strategies [8]. Discrepancies in treatment protocols have also been reported, as the lack of standardization has led to institution-specific management strategies. Many treatment approaches have also mirrored adult BCVI recommendations; limited data support their efficacy in the pediatric population [9]. Due to limited data on patient outcomes, the optimal treatment strategy for pediatric BCVIs has not yet been established. This review examines trends in diagnostic pathways and management strategies, including the accuracy of screening criteria, mechanisms of injury, risk factors, and treatment regimens.

## 2. Materials and Methods

The National Institute of Health National Library of Medicine’s PubMed was queried for studies published from 2010 to 2026. Relevant manuscripts were identified using keywords and MeSH criteria. Search Criteria: ((“((“blunt cerebrovascular injury”[tiab] OR “blunt cerebrovasculartrauma”[tiab] OR”BCVI”[tiab] OR “cervical vascular trauma”[tiab] OR “carotid injury”[tiab] OR “vertebral artery injury”[tiab] OR “carotid artery dissection, traumatic”[MeSH] OR”vertebral artery dissection, traumatic”[MeSH]) AND (“pediatric”[tiab] OR “paediatric”[tiab] OR “child”[MeSH] OR “children”[tiab] OR”infant”[MeSH] OR “adolescent”[MeSH] OR “teenager”[tiab] OR “youth”[tiab]))])) AND (“incidence”[tiab] OR “prevalence”[tiab] OR “frequency”[tiab] OR “epidemiology”[MeSH] OR “etiology”[tiab] OR “mechanism”[tiab] OR “cause”[tiab] OR “trauma”[tiab] OR “motorvehicle accident”[MeSH] OR “diagnosis”[Subheading] OR “screening”[tiab] OR “screeningcriteria”[tiab] OR “Denver criteria”[tiab] OR “Memphis criteria”[tiab] OR “computedtomography angiography”[MeSH] OR “CTA”[tiab] OR “magnetic resonanceangiography”[MeSH] OR “MRA”[tiab] OR “angiography”[tiab] OR “management”[tiab] OR”treatment”[Subheading] OR “therapy”[tiab] OR “anticoagulation”[tiab] OR”antiplatelet”[tiab] OR “stenting”[tiab] OR “surgery”[tiab]))] OR “stroke”[MeSH] OR “cerebralinfarction”[MeSH] OR “ischemic stroke”[tiab]). The final search took place on 16 March 2026, yielding 188 results. Any indexed articles were included in our review for abstract or full-text review.

Manuscripts were included in the full-text review if they met the following criteria: pediatric population (age < 18 years old) and BCVI diagnosis or management (Table 1). Articles were initially screened using the abstracts by two independent authors (NVG and AM) according to the criteria described above. Both authors had to agree to proceed with full-text review. Discrepancies between the authors were taken to an independent third author (NJI) for final review. Full-text reviews of manuscripts were performed in the same fashion as the abstract screening. Manuscripts were excluded from full-text review if the following criteria were present: non-pediatric population (age ≥ 18 years old), no mention of BCVI diagnosis or treatment, review or commentary that did not contain original data, mechanism of injury consisted of penetrating injuries, or inability to access full-text (Table 1). Relevant reviews were included if they consisted of original data in the form of case reports. If a case report had a literature review attached to it, it was decided to only include the case report and disregard the literature review to avoid overlap or duplication. Prevalence, mechanism of injury, screening criteria, diagnostic modality, vascular injuries identified, grading of vascular injury, associated injuries, treatment, and patient risk factors were analyzed. Tables were made in Microsoft excel and figures were made using free-use PRISMA templates or Biorender.

## 3. Results/Discussion

### 3.1. Manuscripts Reviewed

**Figure 1:**
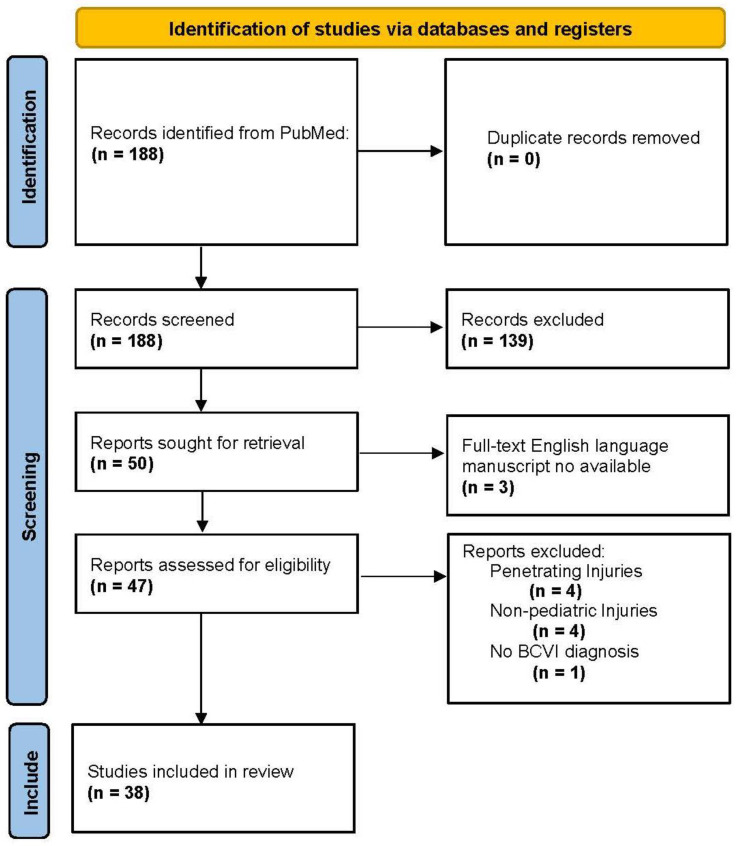
Diagram of pediatric blunt cerebrovascular injury manuscripts selected for review.

Initial search criteria within the PubMed database yielded 188 manuscripts for review. All titles and abstracts were assessed for inclusion, and 50 manuscripts were ultimately selected for full-text review. Three manuscripts were excluded as a full-text English-language manuscript was not accessible. Four manuscripts were excluded due to the inclusion of penetrating injuries in the evaluation of mechanisms of injury. Four manuscripts were excluded from full-text review as they included patients older than 17 years in their studies. One manuscript was excluded due to the absence of BCVI diagnosis. Following the full-text screening, 38 studies were included in the full-text review (Figure 1).

**Figure 2:**
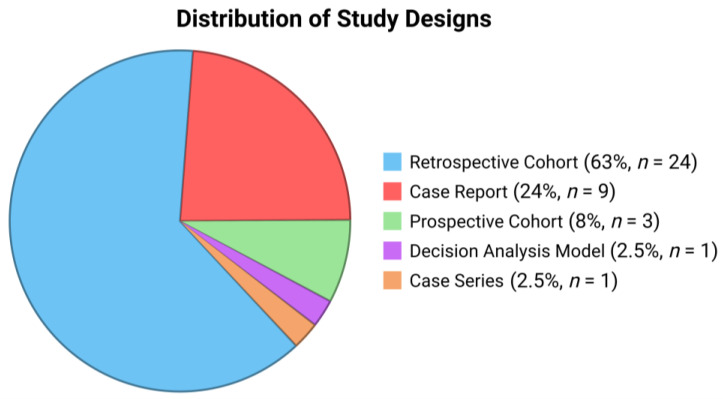
Distribution of study designs among included studies (*n* = 38). Image created with Biorender.

The included studies were predominantly retrospective in nature and included 25 retrospective cohort studies, eight case reports, three prospective cohort studies, one case series, and one decision analytic model (Figure 2). The level of evidence of the queried studies is briefly summarized in Table 2.

### 3.2. Mechanisms of Injury

Motor vehicle collisions (MVCs) represented the most common mechanism of injury (5.9–60% across included studies) resulting in a pediatric BCVI [2,6,9,10,12,13,14,15,16,17,18,21,22,24,25,27,31,32]. The next most common mechanisms of injury include pedestrian vs. automobile (PVA) injuries (7.5–23.5% across included studies) [6,9,10,12,13,14,17,18,25,31,32] and falls (5.9–24.2% across included studies) [9,10,12,13,17,18,32]. While fall height was not uniformly specified across included studies, prior analyses demonstrated a height-dependent relationship between fall height and BCVI risk [10,42]. Strangulation-related mechanisms were less frequent but strongly associated with BCVIs. Approximately 0.9–5.6% of pediatric strangulation cases are diagnosed with a BCVI, much higher than the overall incidence of pediatric BCVIs across all blunt traumatic injuries [6,9,11,25,29]. Other blunt mechanisms, including sports-related collisions, all-terrain vehicle accidents, bicycle crashes, and non-accidental trauma, were also reported but were far less prevalent than those listed above [6,10,12,14,15,16,19,22,31,32,37,39]. Lastly, intraoral and isolated direct neck trauma accounted for only four of the reported cases and were primarily described in case reports [30,33,35,43]. It is important to note that although MVCs, PVAs, and falls were the most prevalent mechanisms of injury, they do not have consistent statistically significant correlations with BCVI diagnosis in our review [2,6,9,10,12,13,14,15,16,17,18,21,22,24,25,27,31,32]. To date, the only mechanism of injury that has shown a consistent statistically significant correlation with BCVI diagnosis in the pediatric population is hanging [11,16,23].

### 3.3. Physical Exam, Associated Injuries, Screening Criteria, and Diagnostic Evaluation

#### 3.3.1. Physical Exam

When evaluating a patient with mechanisms of injury frequently associated with BCVIs, the standard primary and secondary surveys to assess traumatic injury take precedence. Based on the literature, findings from primary and secondary surveys that should prompt further BCVI investigation include: a Glasgow Coma Scale score (GCS) < 8; anisocoria; focal neurological signs; bruit heard in the cervical region upon auscultation of carotids; periorbital hematomas (“racoon eyes”); hemotympanum; cerebrospinal fluid leak; midline cervical tenderness; and readily obvious fractures of the head, face, or neck [2,6,8,9,10,11,12,13,15,16,17,18,21,22,23,24,25,26,27,31,32,35,36,39,41,42]. The above are indicative of TBI, ICH, stroke, carotid artery injury, skull base fracture, cervical spine fracture or ligamentous injury, and head/face/neck fractures respectively; all of these injuries have been strongly associated with pediatric BCVIs and should prompt imaging to evaluate for the presence of a BCVI [2,6,8,9,10,11,12,13,15,16,17,18,21,22,23,24,25,26,27,31,32,35,36,39,41,42]. In contrast, the cervical seatbelt sign has been extensively studied as a marker for screening for BCVIs, yet recent data indicate that its presence alone should not be used as a diagnostic indicator and trigger for imaging due to its low predictive value [23,31].

#### 3.3.2. Associated Injuries

Given the most common mechanisms described in Section 3.2, including MVCs, falls, and PVAs, pediatric BCVI is rarely an isolated finding. High-energy mechanisms predispose patients to have multiple injuries in conjunction with a BCVI. In the papers studied, the most common injuries associated with BCVIs include traumatic brain injury (TBI) and intracranial hemorrhage (ICH), occurring in conjunction with BCVIs in as many as 67% of patients [8,9,13,18,21,31,42]. TBIs were consistently linked to increased injury severities and worse neurological outcomes [8,17,25,42]. Skull base fractures, particularly those involving the carotid canal or petrous temporal bone, are repeatedly identified as strong correlates (17–68% across included studies) of carotid artery injury and intracranial vascular involvement due to the intimate relationship with the associated vasculature [2,9,10,12,13,15,16,21,22,23,25,26,31,32,42,43]. Cervical spine fracture is another commonly associated injury (5.9–26% across included studies), especially in patients with vertebral artery injury [2,6,9,10,12,13,15,16,21,22,23,25,26,27,31,32,42,43]. Although previous studies have not delineated locations within the cervical spine, new studies have specified that upper cervical (C1-C4) fractures or ligamentous injuries are more correlated with BCVIs [21]. Additional injuries reported include facial fractures (Lefort II and III), diffuse axonal injury, thoracic injuries, and high-grade polytraumas characterized by high injury severity scores (ISSs) (>16) [2,10,12,13,26,31,32,39,42].

Isolated BCVIs, without accompanying TBI, ICH, stroke, fractures, or high-ISS injuries, were exceedingly rare in the studies analyzed, with only three reported cases in our study [33,35,36]. Patients in this category usually presented with direct oral blunt trauma or had associated risk factors, such as cervical spine dysplasia, that predisposed them to develop BCVIs from relatively benign mechanisms of injury [33,35,36].

#### 3.3.3. Screening Criteria

In conjunction with the physical exam findings and associated injuries detailed above, numerous screening criteria have been developed to direct clinicians when to obtain imaging, most commonly CTA, for suspected BCVIs. Sensitivities and specificities for screening criteria are calculated using image-confirmed BCVIs as the reference standard, where true positives represent patients with image-confirmed BCVIs who meet the criteria and false negatives represent patients with image-confirmed BCVIs who do not meet the criteria.

The Memphis criteria were initially developed in 2002 for the adult population but has been subsequently used for BCVI screening in the pediatric population [5]. In the Memphis criteria, the presence of the following trigger imaging workup for BCVIs: basilar skull fracture with carotid canal or petrous segment involvement, cervical spine fracture, neurological exam findings not explained by neuroimaging, Horner’s syndrome, facial fractures, and neck soft tissue injury (Table 3). The Memphis criteria demonstrated a high sensitivity (88–91.7%) and an intermediate specificity (71.1–77.5%) in pediatric populations [2,5] (Table 4).

The Utah criteria and McGovern criteria were subsequently developed specifically for the pediatric population in 2017 and 2018, respectively. The Utah criteria consider carotid canal or petrous segment fractures, but include low GCS (<8), focal neurological deficit, and infarct on CT (Table 3) [5,6]. The McGovern criteria are similar to the Utah criteria, with extra consideration for mechanism of injury (MOI) (Table 3) [5,6]. Both Utah and McGovern assign points based on specific risk factors, with a maximum of 11 points possible. A score of ≥3 confers an 18% risk of BCVI and triggers imaging [5,6]. The Utah criteria demonstrated the lowest sensitivity (45.8–52.4%) but the highest specificity (91.3–95.8%) (Table 4) [5,6]. In contrast, in the original multicenter validation studies, the McGovern score demonstrated intermediate specificity (71–89.5%) and intermediate sensitivity (75.0–81%) [5,6]. However, a recent validation study of the McGovern score has demonstrated 90% sensitivity and 96.7% specificity (Table 4) [42].

Based on the limited evidence in the current literature, the Memphis and McGovern criteria have proven to have the highest sensitivities, making them the most appropriate screening measures to minimize the likelihood of missed BCVI in pediatric blunt trauma patients [5,20]. However, the Memphis criteria demonstrated the lowest specificity among all screening tools, potentially increasing unnecessary imaging and radiation exposure [5,20]. Long-term negative consequences of radiation in the pediatric population must be considered due to the high risk of radiation-induced malignancies [44]; therefore, broader screening tools like the Memphis criteria are less favored. In contrast, the Utah criteria have been found to have the highest specificity, leading to fewer patients meeting imaging criteria, decreasing the occurrence of unnecessary radiation exposure [5]. Despite the high specificity, the Utah criteria have a low sensitivity, leading to a higher proportion of undiagnosed children, making this tool’s use unfavorable. Among currently available pediatric-specific screening tools, the McGovern criteria appear to offer a somewhat more favorable balance between sensitivity and specificity, although further external and prospective validation is needed.

As of the most recent query of the literature, new pediatric screening tools for BCVI detection are being developed and validated. The A+ criteria were proposed in 2025 as a novel alternative for pediatric BCVI screening [12]. The A+ criteria delineate temporal, sphenoid, orbital roof fractures and C1-4 ligamentous injuries of the cervical spine as a trigger for imaging [12]. An initial retrospective study demonstrated that the A+ screening criteria could offer a better indication for imaging while potentially delivering a high sensitivity in detecting pediatric BCVIs [12]. However, further prospective validation is needed to define their diagnostic accuracy and formally establish the A+ criteria as an effective screening tool to detect BCVIs in pediatric trauma cases [12].

#### 3.3.4. Diagnostics and Angiographic Grading

Diagnostic modalities used in the evaluation of pediatric BCVIs include Computed Tomography Angiography (CTA), Magnetic Resonance Imaging/Angiography (MRI/MRA), Digital Subtraction Angiography (DSA), and, less commonly, ultrasound. The most commonly used diagnostic modality for pediatric BCVIs is CTA, evidenced by its use as the primary diagnostic method in 78.9–100% of cases [17,45]. CTA is the preferred modality for diagnosis because of its broader accessibility, faster acquisition times, short imaging procedures and rapid image attainment, rendering it particularly useful in acute trauma settings [45]. Adult validation studies have demonstrated CTA sensitivities ranging from 66 to 98% and specificities of 92–100% when compared with Digital Subtraction Angiography (DSA), and while pediatric-specific validation is more limited, available pediatric cohorts suggest comparable diagnostic performance with modern multidetector scanners [46,47,48]. CTA forms the foundation of most pediatric screening algorithms, including those based on structured criteria such as the Denver and McGovern scoring systems mentioned above. Importantly, there are some downsides to CTA use. Notably, CTA has limited sensitivity for detecting BCVIs, which may result in missed injuries and delayed or absent treatment, potentially leading to neurologic complications and associated morbidity [45]. Another downside to using CTA is the known radiation risk; for example, a single head CTA delivers approximately 15–30 mGy to bone marrow, which corresponds to a 1.8-fold increased hematologic cancer risk [49].

MRI/MRA is also commonly used in the diagnosis of pediatric BCVIs; however it is implemented less frequently than CTA [2,5,6,8,9,26,27,42,45]. MRI/MRA, despite having no radiation risk, is limited in its use in the acute trauma setting because of its limited accessibility, long imaging procedure, and frequent need for sedation in the pediatric setting [50]. Furthermore, when compared to CTA, MRI/MRA is outperformed by CTA, with MRA having a sensitivity 50% for carotid injuries and 47% for vertebral injuries [51]. In contrast, when compared to DSA, MRI/MRA has high sensitivity (95%) for diagnosing carotid dissection but a notably lower sensitivity for vertebral artery dissection (60%) [52,53]. MRI/MRA remains a good imaging modality for BCVIs, although it should be employed when the patient is outside of the acute traumatic period or has been stabilized. Furthermore, given the lack of radiation, it may be considered for follow-up imaging in select patients.

DSA is a catheter angiographic study that is considered the historical gold standard, in which CTA and MRI/MRA are imaging modalities used to definitively diagnose or rule out BCVIs [54]. DSA, as an imaging modality, has the highest spatial and temporal resolution for the local vasculature [54]. Despite these benefits, DSA is an invasive procedure in which arterial access is obtained and a catheter is advanced under fluoroscopic guidance to selectively inject contrast material into vessels of interest [55]. Given these characteristics, namely, that DSA is time-intensive, invasive, requires sedation in children, and requires specialized personnel to perform it, it is less useful in the acute trauma setting [55]. Furthermore, DSA carries procedural risks not present in CTA and MRI/MRA, such as groin access complications (4.2%) and iatrogenic cerebral artery injury (1.8%), along with permanent neurological complications in 0.07% of cerebrovascular cases [55]. DSA should be reserved for patients who have equivocal or negative results on CTA or MRI/MRA but high clinical suspicion for BCVI persists.

In contrast, US of the neck was only used in one case for the diagnosis of pediatric BCVI [36]. US has inadequate sensitivity in the diagnosis of BCVI and is user-dependent. Furthermore, imaging of cerebrovasculature is limited by the surrounding bony structures which are in close proximity to the cerebrovascular circulation [56]. In this context, US should only be considered if providers are unable to access CTA, MRI/MRA, or DSA.

**Figure 3:**
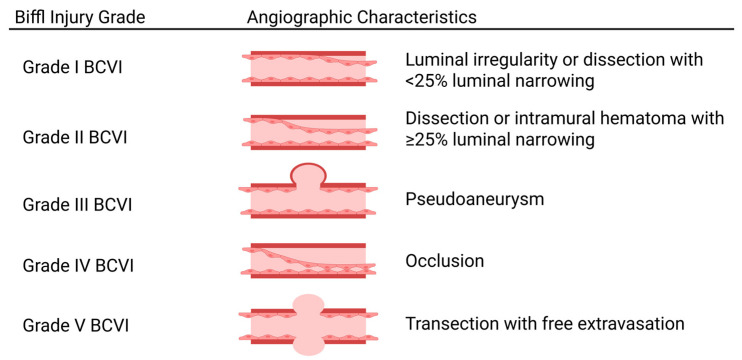
Denver (Biffl) grading scale describing angiographic findings in each grade of BCVI. Figure created using Denver (Biffl) criteria as a reference [57]. Image created with Biorender.

When BCVIs are diagnosed on angiographic imaging, grading is paramount to inform treatment. BCVIs are most commonly graded according to the Denver (Biffl) scale, widely accepted as the gold standard for grading for both carotid and vertebral artery injury (Figure 3) [57]. Grades are assigned from I-V, with V being the most severe form of BCVI. In our study, low-grade injuries, Grades I–II, accounted for approximately 50–75% of cases in large patient cohorts [2,5,6,8,9,16,17,18,21,22,31,32,42,43]. Grade III injuries accounted for 1.7–21% across included studies, while Grade IV injuries represented 4–18%; Grade V injuries were rare, reported in <10% of patients [2,5,6,8,9,16,17,18,21,22,31,32,42,43]. Higher Denver grades were consistently associated with increased stroke risk, whereas mortality correlated more strongly with injury severity score and associated traumatic brain injury [2,5,6,8,9,16,17,18,21,22,31,32,42,43].

### 3.4. Vessels Affected, Treatment, and Outcomes

#### 3.4.1. Vessels Affected

Among the vessels described above, the carotid artery is the most commonly injured in pediatric BCVI, accounting for 27.7–93.8% of BCVIs [23,25]. When injury location was specified, the ICA was the most affected, with both the extracranial and intracranial ICA being affected in similar proportions, accounting for 23–56.3% and 25–75% of injuries, respectively [9,32]. In general, extracranial vessels are more vulnerable to injury because of their relatively fixed proximal and distal attachment points with mobile segments in between, which can increase susceptibility to shear forces at these attachment points. The extracranial ICA is commonly injured because high-energy mechanisms of injury cause the vessel to hyperextend over the lateral articular processes of C1-3 at the base of the skull [43]. Another commonly cited reason for injury is that the extracranial ICA is located laterally within the neck, rendering it more susceptible to injury from direct trauma to the area [43]. The intracranial ICA is commonly affected in pediatric BCVI because it traverses through the petrous and cavernous segments of skull, which are commonly fractured during trauma, a factor previously described as the strongest predictor of BCVI in children [13]. In contrast, the common carotid artery (CCA) is not commonly affected in pediatric BCVI, accounting for approximately 5–10% of carotid injuries [16,31,36,43]. This may be because the CCA is not tethered to adjacent bony structures, such as the skull or vertebrae, allowing for greater mobility during trauma. It is important to note that many of the included studies do not discretely document ICA versus CCA injuries; therefore, CCA injuries may be underreported.

The vertebral arteries (VAs), while less commonly injured than the carotids, still comprise a sizeable portion of affected vessels in pediatric BCVI, accounting for 2.8–57% of injuries [13,24]. When specified, extracranial VAs accounted for 66.7–100% of VA injuries, while intracranial VAs were involved in only 0–33.3% of cases [9,32]. The reason the extracranial VAs are more likely to be affected may be the same as the anatomic reasons listed above for why extracranial carotid injuries are more frequently injured. Specifically, the VA’s V3 segment, located in the C1–C2 region, is a unique anatomical region associated with BCVI because it is tethered to highly mobile cervical vertebrae as it passes through the transverse foramina, which are structures commonly injured during blunt traumas [58]. Conversely, the intracranial VA (V4 segment) is short, mobile, protected in the cranial cavity, surrounded by cerebrospinal fluid, and untethered to bone, making it less prone to injury during blunt trauma [58].

Lastly, the basilar artery (BA) was rarely involved in pediatric BCVI. There were two reported cases of BA injury in our analysis of the literature [34,40]. In one case report, the BA was due to vertebral artery dissection leading to BA thrombosis and occlusion [40]. The second case was a dissection of a BA after a patient fell from a height (~30 ft) and was found incidentally on imaging [34]. The anatomic reasons why the BA is rarely involved are likely similar to the reasons why the intracranial VAs are rarely affected.

#### 3.4.2. Treatment and Associated Complications

Depending on injury severity, patient symptomatology, and institutional guidelines, pediatric BCVI may be managed with observation, medical management (antiplatelet medications or systemic anticoagulation) or surgical management (including endovascular or open approaches). To date, there are no established pediatric-specific treatment guidelines for BCVI, and current management is based on adapted adult protocols with significant practice variation across centers. Overall, antiplatelet or anticoagulation therapy is initiated after the diagnosis of BCVI based on injury stability and extracranial hemorrhage source control [59].

Observation was a common approach for pediatric BCVI, with up to 52% of all pediatric BCVI patients not receiving medical therapy or surgical intervention [8]. Pediatric patients with Grade I BCVIs were more likely to be managed conservatively with observation alone given minimal injury to vessels and the low risk associated with Grade I BCVI [57]. Contrary to this prevailing opinion, recent data have demonstrated a paradoxical relationship between low-grade injuries and a higher risk (2–6.2%) of stroke in patients 0–11 years old [8]. In contrast, higher-grade BCVIs, Grades II-V, represent worse vascular injuries and are therefore less often managed with observation alone [57]. However, many patients with higher-grade BCVIs may present with polytrauma and have contraindications to medical management, warranting observation alone for their BCVIs. Within the current treatment strategies for pediatric BCVI, observation is reserved for patients with contraindication to medical or surgical management or patients with low-grade injury. Its prevalence should not be interpreted as being due to its therapeutic superiority as there have not been prospective studies comparing observation and medical management. If observation is chosen as the modality, Q1H-Q2H neurological checks should be performed while stabilizing the patient with the aim of eventual medical or surgical management.

Barring contraindications, medical management was the preferred strategy for the treatment of pediatric BCVI. In our study, there were changes in medical management according to BCVI grade but not according to the individual vessel injured. When medical management was chosen, antiplatelet therapy was the most frequent medication class used in pediatric BCVI. The most commonly used antiplatelet medication is aspirin [9,27,32,35]. Aspirin is typically used as monotherapy at a pediatric dose of 3–5 mg/kg, with a maximum dosage of 81 mg used for secondary prevention [9,27,32,35]. Other antiplatelet agents used include clopidogrel, which is most used as an alternative to aspirin or an adjunct to aspirin when endovascular stenting is performed [19,32,42]. To date, no studies to our knowledge have been done comparing the efficacy of aspirin and clopidogrel in combination to that of aspirin alone. Multiple studies reviewed show that antiplatelet agents are not inferior to systemic anticoagulation in regard to preventing evolution of neurologic injury in pediatric BCVI [8,32,36]. Antiplatelet agents showed no significant decreases in stroke prevention or hemorrhagic complications and are therefore typically favored in the management of pediatric BCVI [8,32,36], reflecting findings in the adult population, although prospective studies are required to validate these findings. Systemic anticoagulation, such as unfractionated Heparin (UFH) and Low-Molecular-Weight Heparin (LMWH), was used in a smaller subset of patients in the studies reviewed, particularly those with higher-grade BCVI (Grades II–IV) [8,9,16,27,43], likely due to the ability to rapidly reverse it. Systemic anticoagulant use has the benefit of being readily reversible, which may be required in polytrauma patients with high risk of hemorrhage. Furthermore, patients may ultimately be transitioned to an antiplatelet regimen prior to discharge. Other uses for systemic anticoagulation in the management of BCVI include as an adjunct to surgical management, typically in conjunction with endovascular stenting [1].

While rarely required in the pediatric population, surgical approaches are indicated for patients with expanding pseudoaneurysms, thrombosis, or complete occlusions of vessels corresponding to Grade III-V BCVIs [36,38,40]. Endovascular approaches with thrombectomy, stent grafts, or embolization should be considered in patients who have progression of BCVI despite adequate medical management or acute infarcts, if there are no other contraindications [38]. Endovascular approaches are more versatile than open approaches given that there are fewer anatomic constraints, and they could be used to treat CA, VA, and BA injuries. As a result, endovascular approaches were most commonly implemented in the included studies [2,9,13,16,17,18,19,21,38,40,42]. In contrast, open approaches are sparingly used in the management of BCVI, likely due to anatomic constraints and recent advancements in endovascular surgery [9,18,36]. The anatomic location most amenable to an open approach is zone II of the neck (from the cricoid cartilage to the angle of the mandible). Alternatively, zones I and III of the neck may require more invasive approaches, including intrathoracic access via sternotomy/thoracotomy or mandibular subluxation, to adequately obtain vascular control. Open approaches described for the management of BCVI include direct vessel repair for accessible injuries with partial disruption, bypass grafting for injuries requiring vessel reconstruction, or vessel ligation when repair is not feasible and collateral circulation is adequate. However, in this review, the only open approach described in detail was an excision of a pseudoaneurysm at C6 with end-to-end anastomosis [36].

Across studies reporting safety outcomes, treatment-related complications were uncommon. Multiple large database cohorts and large case series reported no hemorrhagic complications attributable to antiplatelet or anticoagulation therapy, and there was very limited reporting of medication adverse events [9,26,42]. Procedural complications were also rarely reported in the small number of endovascular/surgical cases reviewed [2,9,13,16,17,18,19,21,38,40,42]. Therefore, barring contraindications, prompt treatment with antiplatelet agents should be pursued once a BCVI diagnosis is made.

#### 3.4.3. Outcomes

Ischemic stroke is a major driver of morbidity in patients diagnosed with BCVI, occurring in 3.9–37.4% of pediatric patients suffering BCVIs, with those who develop stroke having up to a 33% mortality [8,23,24,25]. Patients less than 11 years old tend to have higher stroke rates (2–6.2%) and lower antithrombotic initiation rates (18.8–22.4%) compared to older pediatric patients (3–4.1% stroke rate and 35.5–63.6% antithrombotic initiation rate) [8].

Aside from stroke, BCVI is an independent risk factor for complicated hospitalization and mortality in the pediatric population [10]. When compared to patients without BCVI, patients with BCVI tend to require intubation at the scene of trauma more often (83.3% vs. 38.8%), need blood transfusions more often (45.7% vs. 10%), and experience more Intensive Care Unit (ICU) and ventilator days (20 d and 15 d vs. 6 d and 3 d) when compared to patients without BCVI [10]. This is likely due to the high prevalence of high-energy mechanisms of injury and severe associated injuries which place patients at high risk for complications.

#### 3.4.4. Follow-Up

Following the prompt evaluation and diagnosis of a BCVI, follow-up imaging was most commonly performed 1–2 weeks after discharge [2,5,12]. While low-radiation modalities such as US and MRI/MRA should be considered, CTA is a prevalent imaging modality performed to evaluate the progression of BCVI. In higher-grade BCVI, the most commonly used follow-up imaging test in our study was CTA, followed by MRA, DSA, and US [2,5,9,32]. After repeat imaging is obtained, clinicians may elect to alter the antiplatelet or anticoagulation regimen depending on interval changes. Generally, if imaging findings are stable or resolved, de-escalation of treatment is warranted [9]. Interval worsening of imaging warrants escalation of medical management and referral for endovascular intervention [9].

### 3.5. Limitations

The presented review has several limitations. Despite a comprehensive search strategy, there is a risk of study selection bias in this review, though strict search criteria, inclusion criteria, and exclusion criteria were adhered to throughout the process to defray this risk. Furthermore, across the full-text studies included in this review, pediatric BCVI cohorts ranged in size from individual case reports to national databases including more than 2000 affected patients. Because several large studies drew from overlapping administrative databases, it was not possible to calculate pooled counts of patients, treatments, and follow-ups to perform a systematic review and meta-analysis. This often resulted in wide ranges for key data points such as vessels involved in BCVIs, stroke, injury patterns, imaging use, and treatment outcomes. These wide ranges likely reflect differences between the varied cohorts in our review and should be interpreted cautiously as they do not represent directly comparable pooled estimates. Further limitations include retrospective analysis of charts using billing codes, which may have led to inadequate capture of granular detail about diagnoses/treatment approaches. In larger database studies, published billing codes were used to identify BCVIs, and specific details on the anatomy and grade of BCVIs and specific treatments were often not recorded. The findings of this review highlight the need for robust prospective studies to determine optimal, screening, diagnostic, and treatment protocols in this population.

## 4. Conclusions

Pediatric blunt cerebrovascular injury (BCVI) represents an uncommon but clinically significant consequence of blunt trauma with a significant risk for ischemic stroke and neurologic morbidity. This review sought to define a general framework for the diagnosis and management of pediatric BCVI using the most contemporary literature. BCVI should be suspected in patients with high-energy mechanisms with findings suggestive of TBI, ICH, basilar skull fractures or cervical spine injuries on primary survey. In such cases, clinicians should use appropriate screening criteria such as the McGovern criteria or others to further inform decisions to escalate imaging. Preferred imaging in diagnosis usually consists of CTA as it is often readily available and amenable for use in the pediatric population. Once BCVI is diagnosed, grading will become crucial to tailor patient-specific treatment. Lower-grade injuries can be managed with single-agent antiplatelet therapy. Higher-grade injuries can be managed with antiplatelet medication, but anticoagulation or surgical interventions should be considered as well. Once patients are discharged, short interval follow-up and low-radiation imaging modalities should be pursued to evaluate injury progression while avoiding excessive radiation exposure. Results of follow-up imaging should be used to inform the next steps in care, whether to keep, escalate or deescalate medical management or pursue surgical interventions. For all patients with these injury patterns, further consideration should be undertaken by clinicians for maintaining flexibility to accommodate local trauma team protocols, multidisciplinary decision-making, and incorporating new studies to further reduce BCVI-related morbidity and mortality.

## Figures and Tables

**Table 1:** Review inclusion and exclusion criteria.

Inclusion Criteria	Exclusion Criteria
Pediatric patient population <18 years old	Non-pediatric patient population ≥18 years old
BCVI diagnosis and/or treatment	No BCVI diagnosis or treatment
	Review or commentary lacking original data
	Penetrating injuries
	Full-text article not accessible in the English language

**Table 2:** Brief summary and levels of evidence of studies queried.

Title	Authors	Study Design	Age Range (Years)	BCVI Cohort Size	Screening Criteria	Diagnostic Modality	Vascular Injuries Identified	Denver (Biffl) Injury Grade	Treatment Strategy	Proportion of Patients with Cerebrovascular Complication	Major Limitations	Reference
Predictors for Pediatric Blunt Cerebrovascular Injury (BCVI): An International Multicenter Analysis	Weber CD, Lefering R, Weber MS, Bier G, Knobe M, Pishnamaz M, Kobbe P, Hildebrand F; TraumaRegister DGU	Retrospective cohort study	0–17	42 patients	Screening criteria not specified	CTA	35 carotid injuries—20 dissections, 5 ruptures/pseudoaneurysms, and 5 bilateral injuries 13 vertebral injuries—5 dissections, 2 transections, 4 occlusions, and 1 bilateral injury	Not reported	Not reported	8.30%	Does not give detailed insights into the onset and course of neurologic symptoms, other than pupil reaction and GCS values; Biffl scale not reported	[10]
Blunt Cerebrovascular Injury in Pediatric Hanging Victims	Golubkova AA, Liebe HL, Leiva TD, Stewart KE, Sarwar Z, Hunter CJ, Johnson JJ	Retrospective cohort study	0–17	10 patients	Screening criteria not specified	CTA	17 carotid artery injuries and 1 vertebral artery injury	Not reported	No antiplatelet prophylaxis reported	0%	Completeness of data reported to database, many charts not filled out completely; Biffl scale not reported	[11]
Pediatric blunt cerebrovascular injury: the McGovern screening score	Herbert JP, Venkataraman SS, Turkmani AH, Zhu L, Kerr ML, Patel RP, Ugalde IT, Fletcher SA, Sandberg DI, Cox CS, Kitagawa RS, Day AL, Shah MN	Retrospective cohort study	0–15	21 patients	McGovern score	CTA (64%), MRA (23%), DSA (1%), and Combination of modalities (11%)	16 carotid artery injuries and 5 vertebral artery injuries	Grade I—4 injuries, Grade II—7 injuries, Grade III—5 injuries, Grade IV—4 injuries, Grade V—1 injury	8/21 patients managed with observation alone; 13/21 patients received medical therapy (12 received antiplatelet and 1 received anticoagulation though it was for the treatment of a deep venous sinus thrombosis)	2/21 patients upon admission and 4/21 patients developed strokes within 24–96 h despite initial antiplatelet therapy	Single-center study	[6]
The A+ criteria for pediatric blunt cerebrovascular injury: An ATOMAC+ multicenter study	Nickoles TA, Eubanks JW 3rd, Lewit RA, Siddique R, Notrica DM, Stottlemyre RL, Ryan M, Johnson J, Maxson RT, Naiditch JA, Lawson KA, Williams R	Prospective, multi-institutional observational study	0–15	25 patients	Memphis criteria	CTA	24 carotid artery injuries and 5 vertebral artery injuries	Not reported	Not reported	20%	Timing of screening and adherence to the screening protocol was not strictly controlled or measured	[12]
Analysis of blunt cerebrovascular injury in pediatric trauma	Grigorian A, Dolich M, Lekawa M, Fujitani RM, Kabutey NK, Kuza CM, Bashir R, Nahmias J	Retrospective cohort study	0–16	109 patients	Screening criteria not specified	Not specified	106 carotid artery injuries and 3 vertebral artery injuries	Not reported	107/109 patients treated nonoperatively and 2/109 underwent endovascular intervention; medical management not specified	2.80%	Does not report Biffl grades of BCVIs and does not indicate which, if any, patients underwent medical therapy	[13]
Analyzing computed tomography Modalities for screening pediatric patients for traumatic blunt cerebrovascular injury	Sainz DB, Howell EC, Grayeb DR, Barlas Y, Gonzalez D, Miskimins R	Retrospective cohort study	0–17	7 patients	1/2 study used expanded Denver criteria and 1/2 study used universal CTA for high-energy blunt traumatic mechanisms	CTA	7 carotid artery injuries	Grade I—6 injuries, Grade II—1 injury	6/7 patients treated with antiplatelet therapy and 1/7 managed with observation alone due to contraindications to antiplatelet therapy	0%	Single-center study and small sample size	[14]
Blunt cerebrovascular injury in children: A prospective multicenter ATOMAC+ study	Lewit RA, Nickoles TA, Williams R, Notrica DM, Stottlemyre RL, Ryan M, Johnson JJ, Naiditch JA, Lawson KA, Maxson RT, Grimes S, Eubanks JW 3rd	Retrospective, multi-institutional observational study	0–14	25 patients	Memphis criteria	CTA (34%), MRA (5%), and DSA (0.2%)	22 carotid artery injuries and 6 vertebral artery injuries	Grade I—14 injuries, Grade II—6 injuries, Grade III—0 injuries, Grade IV—5 injuries, Grade V—1 injury	14/25 patients managed with observation alone (3/14 due to contraindications to therapy), 2/25 patients managed with antiplatelet therapy alone, 2/25 patients managed with anticoagulation alone, 4/25 patients managed with a combination of therapies, and 1/25 patient managed endovascularly	24%	Single-center study with a low compliance with the study protocol screening algorithm	[2]
Risk Factors for Blunt Cerebrovascular Injury in a Cohort of Pediatric Patients With Cervical Seat Belt Sign	Najar DA, Cardenas-Turanzas M, King J, Shah MN, Cox CS, Ugalde IT	Retrospective cohort study	0–17	11 patients	McGovern score	CTA	8 carotid artery injuries and 8 vertebral artery injuries	Grade I—4 injuries, Grade II—4 injuries, Grade III—4 injuries, Grade IV—4 injuries, Grade V—0 injuries	3/11 patients managed with observation alone (1/3 had non-brain death), 5/11 patients managed with antiplatelet therapy alone, 1/11 patients managed with anticoagulation alone, and 2/11 patients managed with a combination of therapies	0%	Prolonged data collection period wherein screening patterns may have changed, single-center study, and small sample size	[15]
Risk Factors in Pediatric Blunt Cervical Vascular Injury and Significance of Seatbelt Sign	Ugalde IT, Claiborne MK, Cardenas-Turanzas M, Shah MN, Langabeer JR 2nd, Patel R	Retrospective cohort study	0–17	53 patients	Screening criteria not specified	CTA	Number of carotid injuries and number of vertebral injuries not specified; 63 cervical vascular lesions identified in total	Grade I—21 injuries, Grade II—14 injuries, Grade III—13 injuries, Grade IV—14 injuries, Grade V—0 injuries	10/53 patients managed with observation alone, 5 patients died, 29/53 patients managed with antiplatelet therapy alone, 7/53 patients managed with anticoagulation alone, 2/53 patients managed with surgery/invasive approach	19%	Prolonged data collection period wherein screening patterns may have changed, single-center study	[16]
Pediatric Versus Adult Blunt Cerebrovascular Injuries: Patients Characteristics, Management, and Outcomes	Asaadi S, Rosenthal MG, Radulescu A, Mukherjee K, Luo-Owen X, Dubose JJ, Tabrizi MB; AAST PROOVIT Study Group	Retrospective cohort study	0–17	38 patients	Screening criteria not specified	CTA (79%) or angiography during intervention	27 carotid artery injuries and 11 vertebral artery injuries	Grade I and II—24 injuries, Grade III—5 injuries, Grade IV—7 injuries, Grade V—2 injuries	11/38 patients managed with observation alone, 19/38 patients managed with antiplatelet therapy alone, 10/38 patients managed with anticoagulation alone, 26/38 managed medically, and 1/38 patients managed with surgery/invasive approach	8%	Unspecified screening criteria and unclear timing and dosage of medical therapy	[17]
Multi-Center Validation of the McGovern Pediatric Blunt Cerebrovascular Injury Screening Score	Venkataraman SS, Herbert JP, Ravindra VM, Yu BN, Bollo RJ, Cox CS Jr, Gannon SR, Limbrick DD Jr, Naftel RP, Ugalde IT, Yorkgitis BK, Weiner HL, Shah MN	Retrospective cohort study	0–15	72 patients	McGovern score	CTA	Number of carotid injuries and number of vertebral injuries not specified	Grade I—34 injuries, Grade II—19 injuries, Grade III—10 injuries, Grade IV—7 injuries, Grade V—2 injuries	33/72 patients managed with observation alone, 26/72 patients managed with antiplatelet therapy alone, 9/72 patients managed with anticoagulation alone (1/9 for management of DVT), and 4/72 patients managed with surgery/invasive approach	19%	Exclusion of patients who underwent MRA or DSA may have led to underestimation of incidence of injuries	[18]
Case Series of Adolescents With Stroke-Like Symptoms Following Head Trauma	Long MK, Arevalo O, Ugalde IT	Case series	14–16	2 patients	Screening criteria not specified	CTA and MRI	2 carotid artery injuries	Not reported	Both patients were managed with an endovascular approach and a combination of antiplatelet and anticoagulant regimens	1	Single-center study and small sample size	[19]
Cost Effectiveness of Pediatric Blunt Cerebrovascular Injury Screening: A Decision Tree Analysis	Campbell AL, Xuan D, Balaraman P, Tatum D, Yorkgitis B, Yu D, McGrew P, Zhang J, Harrell K, Duchesne J, Shi L, Taghavi S	Decision tree analysis	0–17	Not reported	Model compared 7 screening modalities -Denver criteria -Expanded Denver criteria -Memphis criteria -McGovern criteria -Utah criteria -Universal screening -No screening	CTA	Not reported	Not reported	Aspirin is the most cost-effective treatment, though its clinical effectiveness was not evaluated by this analysis	Not reported	Model primarily focuses on cost-effectiveness of approaches to pediatric BCVIs rather than clinical outcomes	[20]
Lower incidence of blunt cerebrovascular injury among young, properly restrained children: An ATOMAC multicenter study	Nickoles TA, Lewit RA, Notrica DM, Ryan M, Johnson J, Maxson RT, Naiditch JA, Lawson KA, Temkit M, Padilla B, Eubanks JW 3rd	Prospective, multi-institutional observational study	0–15	10 patients	Memphis criteria	CTA	9 carotid artery injuries and 4 vertebral artery injuries	Grade I and II—11 injuries, Grade III—0 injuries, Grade IV—1 injury, Grade V—1 injury	Medical therapy not specified, but the patient with a Grade V injury underwent endovascular repair	80%	Small sample size and limited power to evaluate effect of restraints on BCVIs	[21]
Diagnostic accuracy of screening tools for pediatric blunt cerebrovascular injury: An ATOMAC multicenter study	Nickoles TA, Lewit RA, Notrica DM, Ryan M, Johnson J, Maxson RT, Naiditch JA, Lawson KA, Temkit M, Padilla B, Eubanks JW 3rd	Prospective, multi-institutional observational study	0–15	25 patients	Memphis criteria, though data for Denver, EAST, Utah, and McGovern scores were collected	CTA	Number of carotid injuries and number of vertebral injuries not specified	Grade I and II—19 injuries, Grade IV or V—6 injuries	Protocol treatment included systemic anticoagulation for those with multiple injuries and an antiplatelet regimen for those with isolated BCVIs; if the BCVIs had resolved by follow-up CTA at 7–10 days, medical therapy was discontinued, otherwise a neuro-interventional team at each site was consulted	24%	Lack of diagnostic imaging among 86% of the overall cohort may missed clinically silent BCVIs No documentation of arteries affected	[5]
Risk factors for blunt cerebrovascular injury in children: do they mimic those seen in adults?	Kopelman TR, Berardoni NE, O’Neill PJ, Hedayati P, Vail SJ, Pieri PG, Feiz-Erfan I, Pressman MA	Retrospective cohort study	0–14	11 patients	EAST	CTA	9 carotid artery injuries and 2 vertebral artery injuries	Grade I—1 injury, Grade II—9 injuries, Grade III—2 injuries, Grade IV—1 injury, Grade V—0 injuries	6/11 patients managed with observation alone (6 due to contraindications), 2/11 patients managed with antiplatelet therapy alone, and 2/11 patients managed with surgery/invasive approach	38%	Single-center study and small sample size	[22]
Cervical seatbelt sign is not associated with blunt cerebrovascular injury in children: A review of the national trauma databank	Leraas HJ, Kuchibhatla M, Nag UP, Kim J, Ezekian B, Reed CR, Rice HE, Tracy ET, Adibe OO	Retrospective cohort study	0–17	809 patients	Modified Denver and modified Memphis criteria	Not specified	759 carotid artery injuries and 58 vertebral artery injuries	Not reported	Not reported	7%	Unclear imaging and treatment patterns among patients screened	[23]
Pediatric blunt cerebrovascular injuries: A national trauma database study	Savoie KB, Shi J, Wheeler K, Xiang H, Kenney BD	Retrospective cohort study	0–17	1682 patients	Screening criteria not specified	Not specified	791 carotid artery injuries and 957 cerebral artery injuries	Not reported	Not reported	3%	Unclear screening, imaging, and treatment patterns among BCVI patients	[24]
Blunt cerebrovascular injury in pediatric trauma: a national database study	Harris DA, Sorte DE, Lam SK, Carlson AP	Retrospective cohort study	0–20	2150 patients	Screening criteria not specified	Not specified	Carotid artery injuries reported in 28% of cases and vertebral artery injuries in 7% of cases, though most injury locations could not be specified due to coding limitations	Not reported	Medical therapy not specified, but a total of 15 endovascular stenting procedures were performed in this cohort	37%	Unclear screening, imaging, and medical management patterns among BCVI patients	[25]
Blunt cerebrovascular injury in children: underreported or underrecognized?: A multicenter ATOMAC study	Azarakhsh N, Grimes S, Notrica DM, Raines A, Garcia NM, Tuggle DW, Maxson RT, Alder AC, Recicar J, Garcia-Filion P, Greenwell C, Lawson KA, Wan JY, Eubanks JW 3rd	Retrospective cohort study	0–14	23 patients	Memphis criteria	CTA (62%), MRA (38%), DSA (<1%)	21 carotid artery injuries and 6 vertebral artery injuries	Grade I—9 injuries, Grade II—8 injuries, Grade III—2 injuries, Grade IV—4 injuries, Grade V—0 injuries	16/23 patients managed with observation alone, 5/23 patients managed with antiplatelet therapy alone, and 2/23 patients managed with anticoagulation alone	26%	Numerous BCVI patients did not initially meet screening criteria; BCVI rates may be higher than presented	[26]
A cohort study of blunt cerebrovascular injury screening in children: Are they just little adults?	Cook MR, Witt CE, Bonow RH, Bulger EM, Linnau KF, Arbabi S, Robinson BRH, Cuschieri J	Retrospective cohort study	0–17	96 patients	EAST, Denver criteria (DC), and Utah score (US)	CTA (96%) and MRA (4%)	83 internal carotid injuries and 45 vertebral injuries	Grade I—61 injuries, Grade II—34 injuries, Grade III—18 injuries, Grade IV—12 injuries, Grade V—3 injuries	34/96 patients managed with observation alone, 57/96 patients managed with antiplatelet therapy alone, and 1/96 patients managed with an endovascular/invasive approach	18%	Heterogeneity in medical management and underreporting of anticoagulant use	[27]
Implementation of a dual cervical spine and blunt cerebrovascular injury assessment pathway for pediatric trauma patients	Schonenberg Llach M, Fishe JN, Yorkgitis BK	Retrospective cohort study	0–13	3 patients	Denver criteria	CTA	1 carotid artery injury and 2 vertebral artery injuries	Grade II—3 injuries	Not reported	Not reported	Poor adherence to screening/diagnostic protocol; heterogeneity in documenting BCVIs	[28]
Treatment Practices and Outcomes After Blunt Cerebrovascular Injury in Children	Dewan MC, Ravindra VM, Gannon S, Prather CT, Yang GL, Jordan LC, Limbrick D, Jea A, Riva-Cambrin J, Naftel RP	Retrospective cohort study	0–17	52 patients	Discretion of the treating trauma or cerebrovascular team	CTA	47 carotid artery injuries and 10 vertebral artery injuries	Grade I—30 injuries, Grade II—12 injuries, Grade III—5 injuries, Grade IV—2 injuries, Grade V—1 injury	24/52 patients managed with observation alone, 14/52 patients managed with antiplatelet therapy, 8/52 patients managed with anticoagulation, and 4/52 patients managed with open surgery/endovascular approach	31%	Heterogeneous management approaches	[9]
Injury patterns and mortality associated with near-hanging in children	Gorski JK, Smith CM, Ramgopal S	Retrospective observational study	0–17	17 patients	Screening criteria not specified	CTA or MRA	Not reported	Not reported	Not reported	Not reported	Decision to obtain neck angiography unclear, as are management approaches to the BCVIs in this study	[29]
The smallest suffer stroke: Understanding stroke and treatment patterns in children with blunt cerebrovascular injury within the Trauma Quality Improvement Program database	Dawson-Gore CC, Myers EK, Cooper EH, Evans LL, Schauer SG, Acker S	Retrospective cohort study	0–17	2336 patients	Screening criteria not specified	CTA or MRA	Number of carotid injuries and number of vertebral injuries not specified	Grade I and II—1248 injuries, Grade III—40 injuries, Grade IV—412 injuries, Grade V—636 injuries	52% of patients were managed with observation alone, 5% were managed with antiplatelet therapy, and 42% of patients were managed with anticoagulation	4%	Timing of screening and timing of cerebrovascular complications unclear	[8]
Delayed internal carotid artery occlusion and paralysis after oral trauma	Kawakami K, Oyama Y, Watanabe Y, Motoi H, Odaka M, Shiga K, Ito S	Case report	2	1 patient	Screening criteria not specified	CTA and MRA	1 carotid artery injury	Grade IV	Patient managed with an antiplatelet regimen	1/1	Case report with limited generalizability	[30]
Screening CT angiography for pediatric blunt cerebrovascular injury with emphasis on the cervical “seatbelt sign”	Desai NK, Kang J, Chokshi FH	Retrospective cohort study	0–17	8 patients	Screening criteria not specified	CTA	5 carotid artery injuries and 4 vertebral artery injuries	Grade I—1 injury, Grade II—2 injuries, Grade III—1 injury, Grade IV—4 injuries, Grade V—0 injuries	Not reported	25%	Heterogeneity in documenting cervical seatbelt sign and type of physical exam findings constitutes a cervical seatbelt sign	[31]
Comparison of anticoagulation and antiplatelet therapy for treatment of blunt cerebrovascular injury in children <10 years of age: a multicenter retrospective cohort study	Ravindra VM, Bollo RJ, Dewan MC, Riva-Cambrin JK, Tonetti D, Awad AW, Akbari SH, Gannon S, Shannon C, Birkas Y, Limbrick D, Jea A, Naftel RP, Kestle JR, Grandhi R	Retrospective cohort study	0–9	17 patients	Discretion of the multidisciplinary treating team	CTA	15 carotid artery injuries and 3 vertebral artery injuries	Grade I—7 injuries, Grade II—5 injuries, Grade III—1 injury, Grade IV—4 injuries, Grade V—0 injuries	11/17 patients managed with antiplatelet therapy and 6/17 patients managed with anticoagulation	47%	Heterogeneity in BCVI management algorithms and in the reporting of functional outcome measures	[32]
Congenital spine deformities: a new screening indication for blunt cerebrovascular injuries after cervical trauma?	Capone C, Burjonrappa S	Case report	12	1 patient	Screening criteria not specified	CTA and Doppler ultrasound	1 carotid artery injury	Grade II—1 injury	Patient started on systemic anticoagulation then switched to a combination antiplatelet and anticoagulation regimen; patient was ultimately discharged with a 6-month course of an antiplatelet monotherapy	0/1	Case report with limited generalizability	[33]
Isolated basilar artery dissection following blunt trauma challenging the Glasgow coma score: A case report	Moyer JD, Dioguardi Burgio M, Abback PS, Gauss T	Case report	14	1 patient	Screening criteria not specified	CT scan	1 basilar artery injury	Not reported	Contraindications to medical therapy and ultimate brain death	1/1	Case report with limited generalizability	[34]
A case report of blunt intraoral cerebrovascular injury in a child following intraoral trauma: The pen is mightier than the sword	Hon K, Roach D, Dawson J	Case report	5	1 patient	Screening criteria not specified	CTA and Doppler ultrasound	1 carotid artery injury	Grade II—1 injury	Antiplatelet monotherapy	0/1	Case report with limited generalizability	[35]
Blunt cerebrovascular injury: early recognition and treatment options in asymptomatic patient	Becker A, Ashkenazi D, Hershko D	Case report	14	1 patient	Screening criteria not specified	CTA neck	1 carotid artery injury	Grade III—1 injury	Open surgical repair	1/1	Case report with limited generalizability	[36]
Severe Pediatric Polytrauma Complicated by Stroke After Fall From Swamp Buggy	Uebelacker MC, Rago A, Fahmy J, Farish A	Case report	4	1 patient	Screening criteria not specified	CTA	1 carotid artery injury	Grade III—1 injury	Antiplatelet monotherapy	1/1	Case report with limited generalizability	[37]
Treatment of a high large extracranial carotid artery pseudoaneurysm from trauma using a Viabahn graft	David Zaghlool, and Randall Franz	Case report	17	1 patient	Screening criteria not specified	CTA and DSA	1 carotid artery injury	Grade III—1 injury	Anticoagulation and ultimately endovascular stent placement after follow-up imaging demonstrated progression	0/1	Case report with limited generalizability	[38]
Internal carotid artery dissection following blunt head trauma: a pediatric case report and review of the literature	Muhterem Duyu, Selin Yıldız, İrem Bulut, Zeynep Karakaya, Ayşenur Buz, Gülçin Bozbeyoğlu	Case report	14	1 patient	Screening criteria not specified	MRI and CTA	1 carotid artery injury	Not reported	Antiplatelet monotherapy	0/1	Case report with limited generalizability	[39]
Childhood acute basilar artery thrombosis successfully treated with mechanical thrombectomy using stent retrievers: case report and review of the literature	Giancarlo Nicosia, Domenico Cicala, Giuseppe Mirone, Pietro Spennato, Vincenzo Trischitta, Claudio Ruggiero, Gianluigi Guarneri, Mario Muto, Giuseppe Cinalli	Case report	23 (months)	1 patient	Screening criteria not specified	MRA	1 vertebral artery injury	Grade IV—1 injury	Endovascular intervention	1/1	Case report with limited generalizability	[40]
The necessity of CT scans on pediatric carotid injury after blunt trauma-An analysis of the traumaregister DGU	Becker L, Krüger L, Wolf M, Alfen K, Theysohn J, Lefering R, Dudda M, Kamp O; Committee on Emergency Medicine, Intensive Care and Trauma Management (Sektion NIS) of the German Trauma Society (DGU), Germany	Retrospective cohort study	0–15	50 patients	Screening criteria not specified	CT scan	50 carotid artery injuries	Not reported	Not reported	Not reported	Limited reporting of BCVI management and cerebrovascular complications	[41]
Screening Pediatric Trauma Patients for Blunt Cerebrovascular Injury Using the McGovern Score: A Retrospective Cohort Study.	Osorio RG, Johnson AB, Neff LP, Riera KM, Petty JK, Couture DE, Kramer CL, Venkataraman SS, Saha AK, McCrory MC	Retrospective cohort study	0–15	12 patients	McGovern criteria	CTA or MRA (if concurrent neurological deficit)	12 carotid artery injuries and 6 vertebral artery injuries	Grade I—4 injuries, Grade II—5 injuries, Grade III—1 injury, Grade IV—2 injuries, Grade V—0 injuries	6/12 patients managed with observation alone, 3/12 patients managed with antiplatelet therapy, 2/12 patients managed with anticoagulation, and 1/12 patients managed with an endovascular approach	25%	Limited compliance with McGovern criteria	[42]

**Table 3:** BCVI screening criteria.

Screening Tool	Memphis Criteria	Utah Score	McGovern Score
Criteria	Basilar skull fracture with involvement of the carotid canal Basilar skull fracture with involvement of petrous bone Cervical spine fracture Neurological exam not explained by brain imaging Horner’s syndrome Lefort II or III fracture patterns Neck soft tissue injury (seatbelt sign or hanging or hematoma)	GCS score £8 (1pt) Focal neurological deficit (2 pts) Carotid canal fracture (2 pts) Petrous temporal bone fracture (3 pts) Cerebral infarction on CT (3 pts)	GCS score £ 8 (1pt) Focal neurological deficit (2 pts) Carotid canal fracture (2 pts) Mechanism of injury (2 pts) Petrous temporal bone fracture (3 pts) Cerebral infarction on CT (3 pts)
Indication for Imaging	Imaging indicated if any of the above criteria are met	Imaging indicated if patient presents with 3 or more points	Imaging indicated if patient presents with 3 or more points
Year Released	2002	2017	2018
References	[5]	[5,6]	[6]

**Table 4:** Statistical measures of screening criteria.

Screening Criteria	Sensitivity	Specificity	References
McGovern Criteria	75.0–90%	71.3–96.7%	[5,6,42]
Memphis Criteria	88–91.7%	71.1–77.5%	[2,5]
Utah Criteria	45.8–52.4%	91.3–95.8%	[5,6]

## Data Availability

No new data were created or analyzed in this study.

## Abbreviations

The following abbreviations are used in this manuscript:

BA	Basilar Artery
BCVI	Blunt Cerebrovascular Injury
CCA	Common Carotid Artery
CT	Computed Tomography
CTA	Computed Tomography Angiography
DSA	Digital Subtraction Angiography
GCS	Glasgow Coma Scale
ICA	Internal Carotid Artery
ICH	Intracranial Hemorrhage
ICU	Intensive Care Unit
ISS	Injury Severity Scale
LMWH	Low-Molecular-Weight Heparin
MOI	Mechanism of Injury
MRA	Magnetic Resonance Angiography
MRI	Magnetic Resonance Imaging
MVC	Motor Vehicle Collision
PVA	Pedestrian vs. Automobile
TBI	Traumatic Brain Injury
UFH	Unfractionated Heparin
US	Ultrasound
VA	Vertebral Artery
